# Minocycline black bone disease in arthroplasty: a systematic review

**DOI:** 10.1186/s13018-021-02617-w

**Published:** 2021-08-04

**Authors:** William Steadman, Zak Brown, Christopher J. Wall

**Affiliations:** 1 Department of Orthopaedics, Toowoomba Hospital, Pechey Street, Toowoomba, Queensland 4350 Australia; 2 University of Queensland, Toowoomba, Queensland Australia; 3 School of Medicine, Rural Clinical School, University of Queensland, Toowoomba, Queensland Australia

## Abstract

**Background:**

Minocycline black bone disease is a rare finding that can cause concern when unexpectedly encountered during routine arthroplasty. Prolonged minocycline use can cause selective staining of subchondral bone, whilst peri-articular soft tissue and cartilage appear uninvolved.

**Methods:**

A systematic review according to PRISMA guidelines was performed to identify all reported cases in the literature.

**Results:**

Including the patient we present, eleven cases of minocycline black bone disease encountered during arthroplasty have been reported in the literature. All cases have had an excellent outcome, with no complications reported to date.

**Conclusions:**

Minocycline black bone disease can be a concerning intra-operative finding when unexpectedly encountered during routine arthroplasty, but should not affect the operative plan. Surgeons should exclude alternative causes of bone discolouration when the history is unclear.

## Introduction

Discolouration of bone encountered intra-operatively during arthroplasty can be unexpected for the surgeon and raise concerns regarding bone integrity, prosthesis stability and risk of post-operative complications [1]. Despite increasing recognition of minocycline causing bony discolouration, orthopaedic reports remain sparse [2]. Discolouration has been reported as grey, greyish-black, brown, blue-green and black [1, 3].

Minocycline is a synthetic tetracycline derivative, with both antibiotic and anti-inflammatory properties. The latter gives it therapeutic properties in rheumatoid arthritis and dermatological conditions such as acne vulgaris and rosacea. Hyperpigmentation can occur in different body tissues, and minocycline is well-recognised as a cause of bony pigmentation in oral and maxillofacial surgery [2]. Differentials for bony discolouration include ochronosis, metabolic bone diseases, metal deposition including titanium, iron and copper, haemophilic arthropathy, sequestra, metastases and long-term minocycline use [1, 3]. Importantly, all of these conditions tend to stain peri-articular tissues, with the exception of minocycline use [1].

Minocycline is strongly bound to plasma proteins, and very lipid soluble, giving it high tissue and body fluid penetrance [4]. In bone, deposits contain iron and calcium, resembling hemosiderin histologically, but lipofuscin, melanin and minocycline degradation products have been implicated in discolouration [4, 5]. Tetracyclines can chelate calcium and be incorporated into normal bone [1]. Currently, there are no reports of pathological bone in adult patients related to minocycline use, although the process of discolouration is rapid and permanent [3]. Ongoing minocycline use may also cause discolouration of operative scars [6].

Dosage required for bony discolouration remains unclear in the literature. Currently reported cumulative exposures can be seen in Table 1. The minimum reported cumulative dose in arthroplasty literature to cause discolouration is 42.5 g, reported by Yang et al. [7].

Histological diagnosis is performed using standard microscopy, which reveals normal bone structure despite macroscopic discolouration [1, 7, 8]. In contrast, other conditions causing discolouration reveal histologic features of infection, infarction or malignancy, or evidence of heavy metal or homogentisic acid deposition [1, 7, 8]. Haematoxylin and eosin and Berlin blue stains may be used to assess samples histologically [1, 7]. Conflicting reports of the utility of ultraviolet fluorescence exist in the literature and may not be a reliable indicator [3, 8].

A systematic literature review was performed to evaluate current evidence about minocycline black bone disease in patients undergoing arthroplasty, and its effect on clinical outcomes.

## Case report

A 52-year-old female presented to the orthopaedic outpatient clinic with severe, activity-related, bilateral knee pain, worse on the right side. Clinical examination of her right knee demonstrated medial joint line tenderness, decreased range of motion (ROM) of 15 to 80 degrees, and intact collateral and cruciate ligaments. Radiographs demonstrated advanced tricompartmental osteoarthritis (OA), with complete joint space loss in all compartments.

The patient’s past medical history included angina, asthma, gastro-oesophageal reflux disease, and cigarette smoking. The patient had been taking minocycline to treat acne vulgaris for 32 years at 50 mg per day, prior to her initial operation (total exposure of approximately 584 g).

The patient had exhausted non-operative treatment for her knee pain. Given that her right knee was clinically and radiographically worse than her left, she was booked for a right total knee arthroplasty (TKA).

Intra-operatively, black staining of the subchondral bone was noted, with no involvement of peri-articular soft tissues or remaining cartilage. The findings were thought to be related to minocycline use and bone specimens were sent for histopathology. TKA was performed using navigation. The patella was resurfaced due to full thickness chondral loss. All components were cemented.

**Fig. 1:**
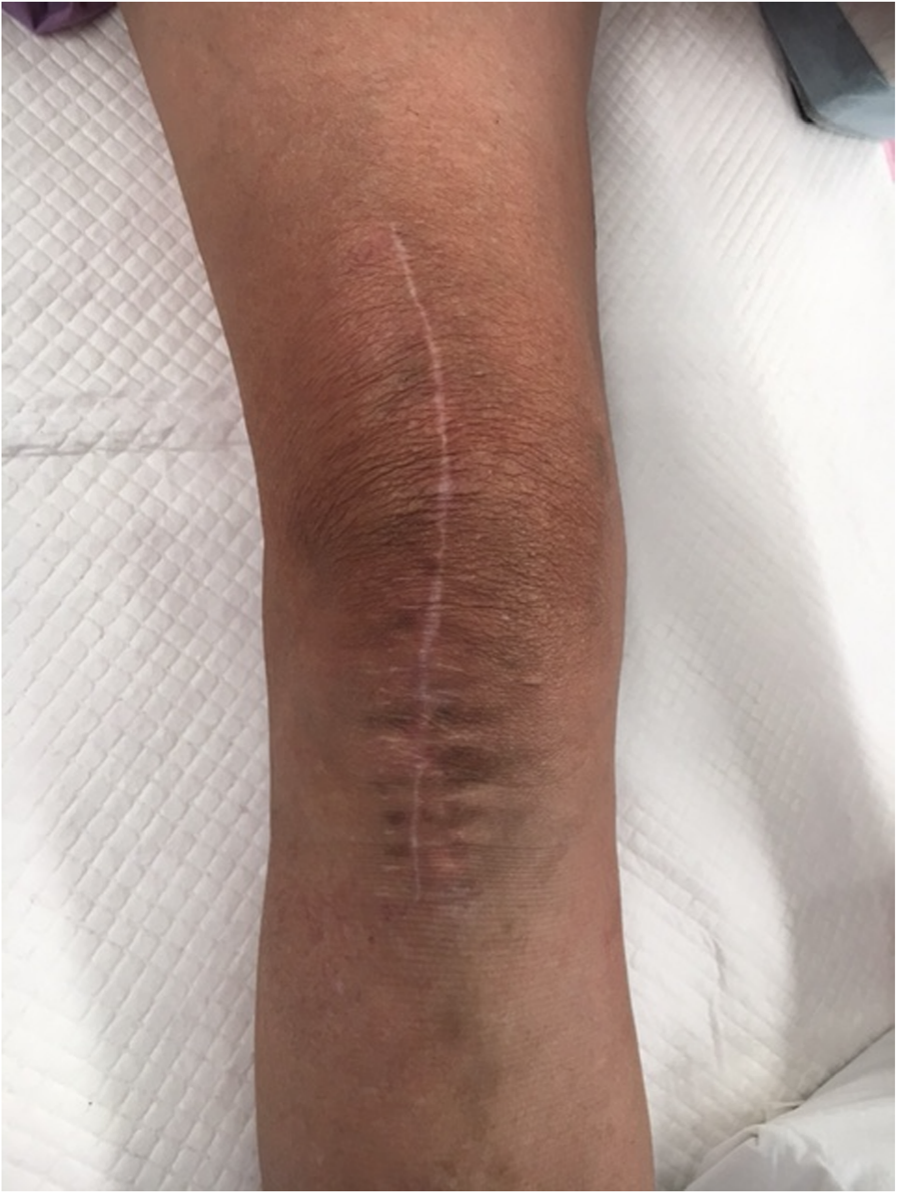
Operative scar following right total knee arthroplasty

Histology of bone and synovial tissue was consistent with OA, with no other concerning features. The patient made an uneventful recovery. At 12-month review, the patient was pain free with 0 to 130 degree ROM and independent mobility. Slight skin discolouration was noted around the operative scar (Fig. 1). Post-operative radiographs were unremarkable.

The patient complained of worsening left knee pain despite appropriate non-operative treatment. Clinical examination demonstrated mild varus alignment with a limited ROM of 20 to 90 degrees. Radiographs revealed advanced medial compartment OA, with complete joint space loss.

**Fig. 2:**
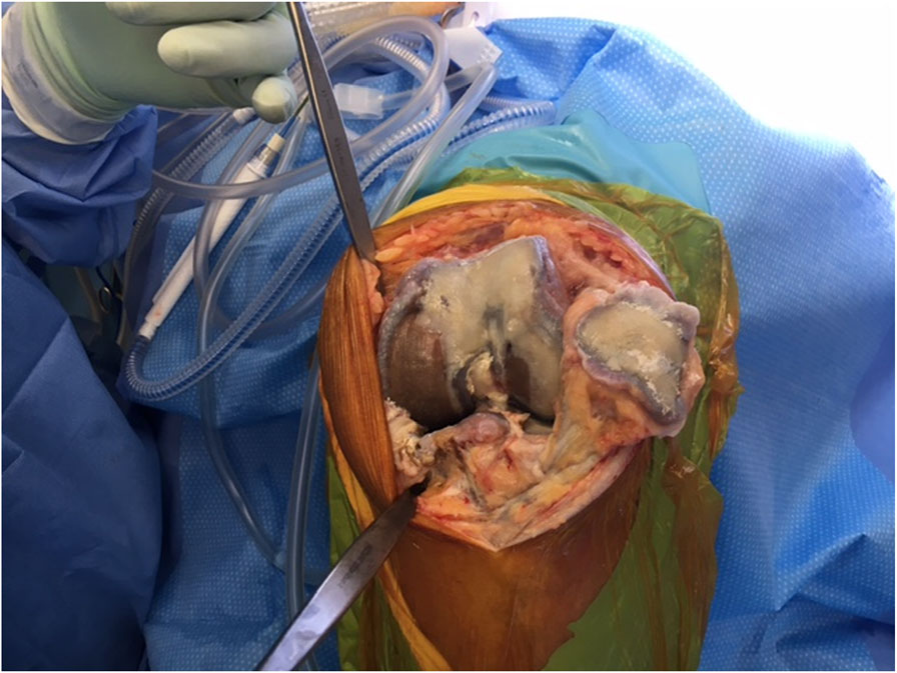
Femoral surface

**Fig. 3:**
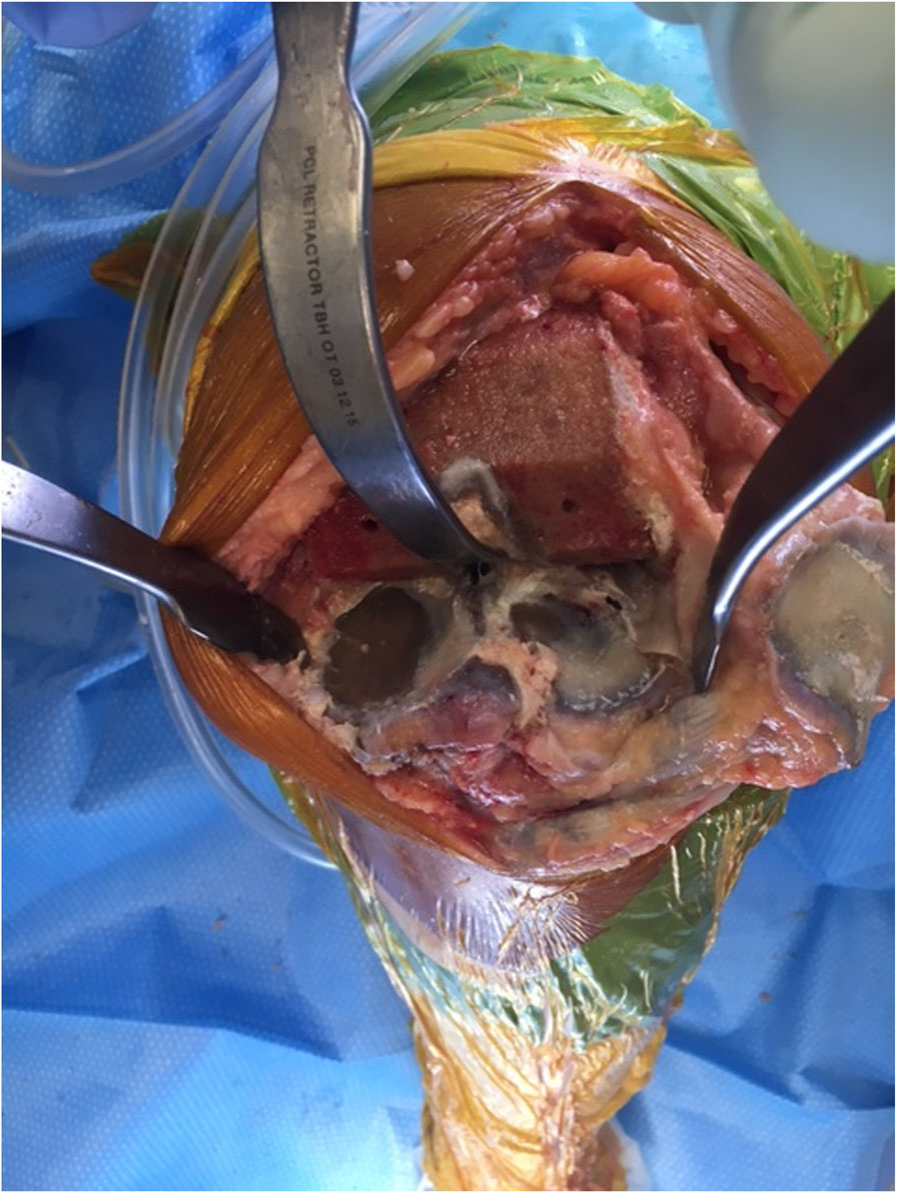
Tibial surface

The patient underwent a left TKA 2 years after her contralateral procedure. Black bony discolouration was again noted, with normal peri-articular soft tissues and remaining cartilage (Figs. 2 and 3). TKA was performed using navigation. Patellar osteophytes were removed but the patella was not resurfaced as the cartilage was intact. Both components were cemented. Histopathological examination of the bone was unremarkable. The patient made an uneventful recovery. At 12-month review, the patient was pain-free and very satisfied, having returned to full function with ROM of 0 to 120 degrees and a stable knee. Post-operative radiographs were unremarkable.

At final review, 4 years post right TKA and 2 years post left TKA, the patient remained pain free with an excellent functional outcome. Radiographs did not demonstrate any evidence of component loosening or migration.

## Materials and methods

In accordance with Preferred Reporting Items for Systematic Reviews and Meta-Analyses (PRISMA) guidelines, a systematic literature search of MEDLINE, Embase and Cochrane databases was conducted for articles relevant to minocycline bone disease in arthroplasty, on April 15, 2020 [9]. No restrictions were placed on the search, including language or publication year. Search terms included (“MINOCYCLINE” AND (“ARTHROPLASTY” OR “ARTHOPLASTIES” OR “TOTAL JOINT” OR “JOINT REPLACEMENT” OR “PROSTHESIS IMPLANTATION” OR “JOINT PROSTHESIS” OR “TOTAL ELBOW REPLACEMENT” OR “TOTAL SHOULDER REPLACEMENT” OR “FINGER REPLACEMENT” OR “TOTAL HIP REPLACEMENT” OR “TOTAL KNEE REPLACEMENT” OR “TOTAL ANKLE REPLACEMENT”)). Additional articles were found through manual review of literature and references of relevant articles. Full-text articles were manually screened for relevance by two independent reviewers (W.S. and Z.B.), and discrepancies were discussed until agreed upon.

## Results

**Fig. 4:**
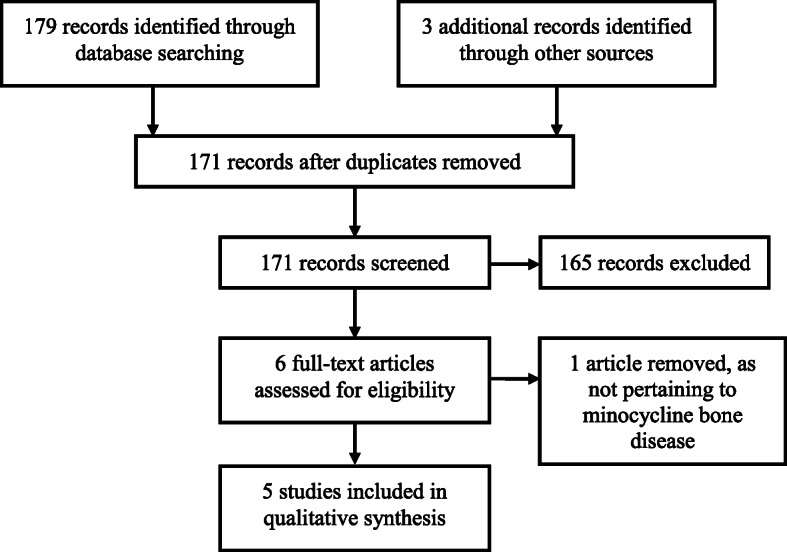
PRISMA flowchart

A total of 179 articles were found through the structured database search, six were identified for full-text review, and five were included in the article (Fig. 4). All reported cases of minocycline bone disease in arthroplasty, with pre- and post-operative data available are listed in Table 1. A single case series was found, along with four case reports. Data about patient demographics, procedure, intra-operative findings, histology and clinical and radiographic outcomes was extracted.

**Table 1:** Reported Cases

Study	Age	Joint	Indication	Fixation	Components	Subchondral bone	Minocycline dose (cumulative, g)	Follow-up (years)	Outcome—pain	Outcome—function
Ashukem et al. (2016)	Not noted	Primary shoulder	OA	Uncemented stem, cemented glenoid	Press-fit humeral stem, cemented pegged glenoid (DJO Turon)	Greyish-black	*Unspecified*	0.5	Little to no pain	Normal ADLs, return of ROM, stable shoulder
Chauhan and McDougall (2014)	82	Primary knee (bilateral)	OA			Black, normal cancellous bone				
McCleskey and Littleton (2004)	81	Primary knee (right)	OA	Cemented	*Unspecified*	Blue-green-grey	~73	1		Return of ROM, stable knee
Reed, Gregg, and Corpe (2012)	55	Primary knee (left)	OA	Cemented	Not noted, patella not resurfaced	Black, normal cancellous bone	> 365	0.25	No pain	Normal ADLs, return of ROM, stable knee
Yang et al. (2012)	75	Primary ankle	Reactive arthritis		Fine total ankle	Black	140	4.6	Occasional arthritic pain	Normal ADLs
	49	Primary knee	RA		Biomet Vanguard	Black	131	2.8	No pain	Normal ADLs
	77	Revision knee	OA		Zimmer Nexgen LCCK	Black	176	2	Not noted—multiple cerebral infarctions at 2 years	
	48	Primary hip	RA		Zimmer ZCA cup and CPT stem	Black	94.3	3.8	No pain	Normal ADLs
	33	Revision hip	Sarcoidosis		Zimmer ZCA cup with JMM KT plate, Zimmer CPT stem	Black	42.5	3.6	No pain	Normal ADLs
This study (2021)	57	Primary knee (right)	OA	Cemented	Stryker Triathlon PS, patella resurfaced	Black	584	4	No pain	Normal ADLs, return of ROM, stable knee
	59	Primary knee (left)	OA	Cemented	Stryker Triathlon CR, patella not resurfaced	Black	621	2	No pain	Normal ADLs, return of ROM, stable knee

Notably, all reported cases had minocycline-related discolouration confirmed on histology. All cases had no discolouration of peri-articular soft-tissues or remaining cartilage, and all had good radiographic alignment and stability on follow-up. Unfortunately, Chauhan et al. did not report on any of these data points.

## Discussion

Minocycline black bone disease is a rare condition that may be encountered unexpectedly during arthroplasty. Based on the findings of this study, surgeons can be reassured that good clinical outcomes can be achieved with routine arthroplasty, and there is no need to alter the operative plan [2]. Other causes of bone discolouration need to be excluded, but sparing of the cartilage and peri-articular soft tissues appears to be indicative of minocycline bone disease [1]. Histopathological evaluation of bone is recommended, as other diagnoses may have significant impacts on post-operative care.

Costal cartilage has previously been reported as darkened with prolonged minocycline use, but no reports of cartilage discolouration exist in the setting of arthroplasty [10]. Peri-articular soft tissues do not appear to be affected by minocycline bone disease. Extra-articular soft tissue discolouration has been previously reported in relation to minocycline use; however, this has not been reported in arthroplasty [4].

When encountering pigmented bone, the orthopaedic surgeon should be aware of potential diagnoses and their associated clinical features to ensure that appropriate steps are taken intra-operatively. Patients with ochronosis develop joint degeneration following cartilaginous erosion, with pigmentation of cartilage, bone and connective tissue. Bony architecture is unchanged [11] and good patient outcomes following TKA have been reported in the literature, but increased risk of osteopenia has been demonstrated [12, 13]. Heavy metal deposition may be suspected due to medical or occupational history, and serum levels of titanium, iron or copper may be confirmatory. Haemophilic arthropathy may cause early joint degeneration, but this is likely to be clear from the medical history. Infection may also be suspected in the presence of stained tissue, especially with sequestra. A full workup for osteomyelitis is indicated with fresh samples sent for culture. This diagnosis is likely to necessitate a change in operative plan due to the significant risk of subsequent prosthetic joint infection. A metastatic process may also require adjustment of operative plan, with increased concern for implant stability or tumour spread. In patients without a previous diagnosis, a tissue biopsy should be performed. Overall, it is important for the orthopaedic surgeon to perform a thorough history and clinical examination, and order appropriate investigations pre-operatively, to ensure this information is available for diagnostic purposes at the time of surgery.

There are limitations to the conclusions that can be drawn from the current body of evidence, as no studies exist at a higher level than case series. Our findings are at significant risk of publication bias, as surgeons may not be willing to publish reports of cases that had a suboptimal outcome. Further reporting of cases will help to determine the prevalence of minocycline black bone disease and the longer-term outcomes post arthroplasty. Reporting of patients’ total minocycline exposure may help to find the level at which bony discolouration occurs.

Minocycline black bone disease is a rare finding that can cause concern when unexpectedly encountered during routine arthroplasty. Currently, no reports exist of poor outcomes in the presence of this disease; however, surgeons should exclude alternative causes of bone discolouration when the history is unclear. Selective discolouration of subchondral bone with sparing of peri-articular soft tissues and cartilage may be a reassuring clinical sign of minocycline bone disease; however, this requires further investigation.

## Data Availability

Not applicable

## Abbreviations

ROM: Range of motion
OA: Osteoarthritis
TKA: Total knee arthroplasty
PRISMA: Preferred Reporting Items for Systematic Reviews and Meta-Analyses (PRISMA)
