# Correction to: Minocycline black bone disease in arthroplasty: a systematic review

**DOI:** 10.1186/s13018-022-03183-5

**Published:** 2022-06-03

**Authors:** William Steadman, Zak Brown, Christopher J. Wall

**Affiliations:** 1 Department of Orthopaedics, Toowoomba Hospital, Pechey Street, Toowoomba, QLD 4350 Australia; 2 University of Queensland, Toowoomba, QLD Australia; 3 School of Medicine, Rural Clinical School, University of Queensland, Toowoomba, QLD Australia

## Correction to: Journal of Orthopaedic Surgery and Research (2021) 16:479 10.1186/s13018-021-02617-w

Following publication of the original article [1], the author has added the new information in the funding information.

“A Toowoomba Hospital Foundation & Pure Land Learning College Research Grant was received following conclusion of this work to aid in publication.”

The original article has been revised.
